# The association between transient childhood psychotic experiences and psychosocial outcomes in young adulthood: examining the role of mental disorder and attachment

**DOI:** 10.1192/j.eurpsy.2022.1089

**Published:** 2022-09-01

**Authors:** L. Staines, C. Healy, D. Cotter, I. Kelleher, M. Cannon

**Affiliations:** Royal College of Surgeons in Ireland, Psychiatry, Dublin, Ireland

**Keywords:** psychotic experiences, youth mental health, early intervention, mental health outcomes

## Abstract

**Introduction:**

Psychotic experiences (PE) occur most often in childhood, at the same age many mental disorders (MD) develop. There is growing evidence that those who report PE and MD show poorer health outcomes. If this occurs in psychosocial outcomes e.g. self-esteem, stress, mental distress, or social support, is under examined. Attachment anxiety and avoidance are the dimensions of attachment, which is hypothesized to develop in infancy as a mechanism for interpersonal relationships in times of need.

**Objectives:**

To examine the role of transient childhood PE in adult psychosocial outcomes, in those with and without MD. Additionally, to examine if the dimensions of attachment attenuate this model.

**Methods:**

One hundred and three participants attended baseline (age 11 – 13) and 10-year follow-up. PE and MD were collected using the Schedule for Affective Disorders and Schizophrenia for School-aged Children, Present & Lifetime Version. Attachment and outcomes were collected using self-report measures. Analysis compared those with PE, MD and PE and MD, to healthy controls.

**Results:**

PE in childhood was associated with lower self-esteem and lower perceived social support from friends. Lower self-esteem in adulthood was more pronounced in those reporting PE and MD, and was additionally associated with stress in relationships, daily life, and mental distress. Childhood MD without PE was not significantly associated with any psychosocial outcomes. Attachment dimensions significantly attenuated the relationship between PE and self-esteem.

**Conclusions:**

This paper illustrates the significant association of childhood PE on adult outcomes, independent of the effect of co-occurring MD, and demonstrate attachment dimensions role in this model.

**Disclosure:**

No significant relationships.

